# Physical and biological beam modeling for carbon beam scanning at Osaka Heavy Ion Therapy Center

**DOI:** 10.1002/acm2.13262

**Published:** 2021-05-16

**Authors:** Shinichiro Fujitaka, Yusuke Fujii, Hideaki Nihongi, Satoshi Nakayama, Masaaki Takashina, Noriaki Hamatani, Toshiro Tsubouchi, Masashi Yagi, Kazumasa Minami, Kazuhiko Ogawa, Junetsu Mizoe, Tatsuaki Kanai

**Affiliations:** ^1^ Hitachi, Ltd. Research & Development Group Hitachi‐shi Ibaraki Japan; ^2^ Hitachi, Ltd. Smart Life Business Management Division Kashiwa‐shi Chiba Japan; ^3^ Osaka Heavy Ion Therapy Center Osaka‐shi Osaka Japan; ^4^ Department of Carbon Ion Radiotherapy Osaka University Graduate School of Medicine Suita‐shi Osaka Japan; ^5^ Department of Radiation Oncology Osaka University Graduate School of Medicine Suita‐shi Osaka Japan

**Keywords:** beam modeling, carbon beam scanning, LQ model, RBE, triple Gaussian

## Abstract

We have developed physical and biological beam modeling for carbon scanning therapy at the Osaka Heavy Ion Therapy Center (Osaka HIMAK). Carbon beam scanning irradiation is based on continuous carbon beam scanning, which adopts hybrid energy changes using both accelerator energy changes and binary range shifters in the nozzles. The physical dose calculation is based on a triple Gaussian pencil‐beam algorithm, and we thus developed a beam modeling method using dose measurements and Monte Carlo simulation for the triple Gaussian. We exploited a biological model based on a conventional linear‐quadratic (LQ) model and the photon equivalent dose, without considering the dose dependency of the relative biological effectiveness (RBE), to fully comply with the carbon passive dose distribution using a ridge filter. We extended a passive ridge‐filter design method, in which carbon and helium LQ parameters are applied to carbon and fragment isotopes, respectively, to carbon scanning treatment. We then obtained radiation quality data, such as the linear energy transfer (LET) and LQ parameters, by Monte Carlo simulation. The physical dose was verified to agree with measurements to within ±2% for various patterns of volume irradiation. Furthermore, the RBE in the middle of a spread‐out Bragg peak (SOBP) reproduced that from passive dose distribution results to within ±1.5%. The developed carbon beam modeling and dose calculation program was successfully applied in clinical use at Osaka HIMAK.

## 1 INTRODUCTION

Carbon therapy has been conducted for more than 20 years in Japan and Germany. Excellent therapeutic results have been obtained by taking advantage of its high physical dose concentration and high biological effect. It has been proven to have advantages over conventional photon or proton therapy for treating tumors that are highly resistant to radiation, such as those with hypoxia. The excellent clinical results for carbon therapy have led to construction plans for more carbon therapy facilities worldwide, and scanning irradiation has recently been attracting attention among those interested in carbon therapy. In Japan, the National Institute of Radiological Sciences (NIRS) started investigating carbon beam scanning therapy in 2011 and later introduced a superconducting rotating gantry equipped with a scanning system into clinical use. The Kanagawa facility has also started carbon therapy based on scanning irradiation. In addition, the basic design and construction of the Osaka Heavy Ion Therapy Center (Osaka HIMAK) started in 2014, and clinical commissioning, including beam modeling for treatment planning, started in 2018.

Carbon scanning treatment planning requires precise beam modeling with absolute dose calculation to determine the number of carbon particles irradiated to every spot. Moreover, a carbon beam has high biological effect around the Bragg peak, so cell killing of tumors and normal tissues must be controlled by assuming an appropriate biological model. In starting carbon scanning treatment at NIRS, a biological model based on a microdosimetric kinetic model (MKM) was proposed.^1^, ^2^ In addition to the MKM, an updated clinical dose system adopting a carbon beam as standard radiation and considering the dose dependency of the relative biological effectiveness (RBE) was also introduced.^3^

In contrast with the above trend, this study is rather conservative with respect to the biological model. We instead use a well‐proven biological model established by passive carbon irradiation, which is based on a conventional linear‐quadratic (LQ) model and the theory of mixed radiation fields with different radiation quality.^4^, ^5^ This approach fully complies with the carbon dose distribution accumulated so far for passive irradiation, on which a dose fractionation and escalation study was based.^6^ The biological and clinical doses for normal tissue vary significantly by introducing the RBE dose dependency, however, and this means that the normal tissue complication data accumulated in past carbon broad‐beam therapy can no longer be used. In starting carbon scanning therapy at Osaka HIMAK, we thus consider it too early to introduce the RBE dose dependency, when a comprehensive conclusion as to the dose level for normal tissue complication has not yet been achieved. Therefore, we still use the photon equivalent dose to describe the biological and clinical doses, and we fix the RBE at 10% HSG survival without dose dependency to evaluate the clinical dose for normal tissue in a compatible way.

The aim of this study is to develop physical and biological dose modeling for carbon scanning treatment planning. The treatment planning software is VQA Plan (Hitachi, Ltd.). To apply this software to clinical use, we registered appropriate carbon beam scanning data reflecting the characteristics of the irradiation nozzle, and radiation quality data such as the linear energy transfer (LET) needed to calculate biological effects. Then, the biological dose optimization and dose calculation function were implemented in VQA Plan.

## 2 MATERIALS AND METHODS

### 2.A Physical dose modeling

#### 2.A.1 Irradiation apparatus and beam model

**Fig. 1:**
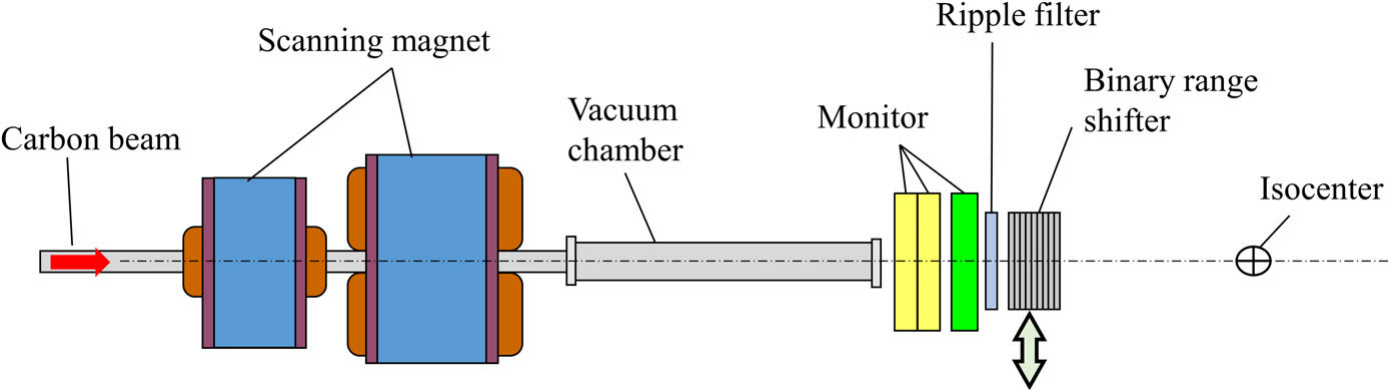
Carbon scanning irradiation nozzle at Osaka HIMAK.

Figure 1 shows the irradiation nozzle at the Osaka HIMAK facility. The facility has three treatment rooms, of which two have two fixed irradiation ports with vertical and horizontal incidence, while one has two fixed ports with horizontal and oblique (45°) incidence. A carbon beam accelerated to the range of 100 to 430 MeV/n by a synchrotron accelerator is transported to the irradiation nozzles and scanned in the x and y directions by scanning magnets. A vacuum chamber extends upstream of the beam monitors, and its distance from the isocenter is about 1.4 m. Of the three installed beam monitors, two are ionization chambers to measure the irradiated particle number of the carbon beam, and one is a multi‐wire proportional counter to detect the beam position in the x and y directions. The carbon scanning beams are prepared at a 3‐mm range interval, and the beam energy is changed by adjusting both the accelerator energy and the binary range shifters in the nozzles. The pristine carbon Bragg peaks are broadened by a ripple filter created by a 3D printing technique. The accelerator provides 12 energies from 100 to 430 MeV/n, and the range shifters provide beams of intermediate energy. The irradiation scheme uses continuous beam scanning, in which the carbon beam is continuously extracted from the synchrotron and irradiated spot by spot without turning the beam off and on. The beam intensity can be adjusted to a constant ranging from 1 to 10 MU/s by tuning the extraction RF power of the synchrotron to specify the intermediate dose between spots when the continuous beam is moved from one spot to the next. Note that MU stands for “monitor unit,” which corresponds to the particle number of the carbon beam and is defined later in the text.

The dose calculation algorithm for carbon treatment planning is based on a triple Gaussian model^7^ :

(1) $$d_{i}=\sum_{j}d_{\mathrm{ij}}w_{j}\equiv \sum_{j}\left({d^{\left(1\right)}}_{\mathrm{ij}}+{d^{\left(2\right)}}_{\mathrm{ij}}+{d^{\left(3\right)}}_{\mathrm{ij}}\right){\;w}_{j},$$

where *d_i_* denotes the physical dose at calculation point (or voxel) *i*, and *d_ij_* is the dose contribution from beam *j* to point *i*, and *w_j_* is the particle number of carbon beam *j* in terms of MU. The physical dose *d_i_* at point *i* is thus calculated by summing over three *d_ij_* components, where the small upper indices 1, 2, and 3 denote the respective dose contributions from the first, second, and third components of the pencil beam. The physical dose contributions *d_ij_^(1)^, d_ij_^(2)^,* and *d_ij_^(3)^* are calculated by multiplying the integral depth dose (IDD) by the lateral Gaussian distribution:

(2) $${d^{\left(n\right)}}_{\mathrm{ij}}={\mathrm{IDD}}_{n}\left(z\right)\times G_{n}\left(z\right)\;\;\;\;\;\;\;\;\left(n=1,\;2,\;3\right).$$

Here, *IDD_n_(z)* denotes the IDD of the n‐th component, and *G_n_(z)* is the lateral two‐dimensional Gaussian distribution at depth *z*:

(3) $$G_{1}\left(z\right)=\frac{1}{2\pi \sigma_{1,x}\left(z\right)\sigma_{1,y}\left(z\right)}\exp\left[‐\frac{{\left(x_{i}‐x_{j}\left(z\right)\right)}^{2}}{2{\sigma_{1,x}\left(z\right)}^{2}}\right]\exp\left[‐\frac{{\left(y_{i}‐y_{j}\left(z\right)\right)}^{2}}{2{\sigma_{1,y}\left(z\right)}^{2}}\right],$$

(4) $$G_{n}\left(z\right)=\frac{1}{2\pi {\sigma_{n}\left(z\right)}^{2}}\exp\left[‐\frac{{\left(x_{i}‐x_{j}\left(z\right)\right)}^{2}+{\left(y_{i}‐y_{j}\left(z\right)\right)}^{2}}{2{\sigma_{n}\left(z\right)}^{2}}\right]\;\;\;\;\;\;\left(n=2,\;3\right),$$

where *σ_1,x_(z), σ_1,y_(z), σ_n_(z) (n = 2,3)* are the beam sizes at depth *z*, *(x_i_, y_i_)* is the lateral position of point *i* and, *(x_j_(z), y_j_(z))* is the center position of beam *j* at depth *z*. The first component in Eqs. (1), (2), and (3) corresponds to the incident ^12^C particles, and the beam sizes in the *x* and *y* directions, *σ_1,x_(z), σ_1,y_(z)*, are calculated by considering the beam’s optical parameters and multiple coulomb scattering in the body. The second and third components in Eqs. (1), (2), and (4) correspond to the fragment halo with larger beam sizes than for the first component. In that case, the beam sizes *σ_2_(z), σ_3_(z)* are isotropic in the x and y directions, as determined from the frame pattern irradiation measurements described in Section 2.A.4. Regarding the triple Gaussian beam model expressed in Eqs. (1), (2), (3), and (4), *IDD_n_(z) (n = 1,2,3)* in the depth direction and the beam sizes *σ_1,x_(z), σ_1,y_(z), σ_n_(z) (n =* 2,3*)* in the lateral direction are parameters to be determined in the physical beam‐modeling process.

**Fig. 2:**
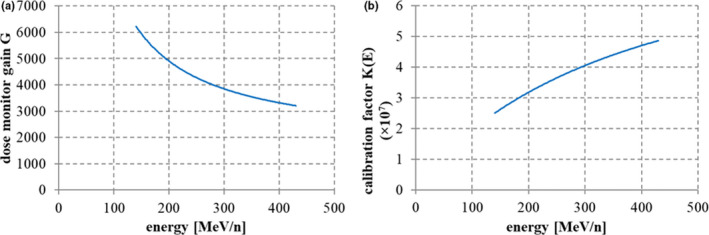
(a) Dose monitor gain *G* with a gap size of 1 cm, and (b) calibration factor *K(E)* (number of carbon particles per MU).

Here, we introduce MU units to describe the irradiated particle number of the carbon beam, as follows. In the ionization dose monitor, electrodes collect ion pairs generated by the carbon beam’s passage through the air, and then electric circuits convert the accumulated charge into a pulse signal. We introduce the gain *G* of the dose monitor, ie the number of ion pairs generated by one incident carbon particle, as

(5) $$G=\frac{\frac{1}{\rho }{\left.\frac{\mathrm{dE}}{\mathrm{dx}}\right|}_{\mathrm{air}}\times g\times d}{W},$$

where *1/ρdE/dx|_air_* denotes the stopping power of ^12^C in air, *g* is the dose monitor’s air gap, *d* is the density of air, and *W* denotes the air’s W‐value to generate one ion pair (35.1 eV). Figure 2a shows the design value of the gain *G* for the case of a 1‐cm electrode gap *g* in the monitor. We chose an ionization amount of 25 nC as 1 MU so that about 100 MU would correspond to irradiating a physical dose of 1 Gy in a 1‐liter volume, as described in 8. In this definition, the calibration factor *K(E)* of the dose monitor,^9^ ie the number of carbon particles per MU, is calculated as

(6) $$K\left(E\right)=\frac{N}{\mathrm{MU}}=\frac{25[\text{nC}]}{G\times e},$$

where *e* denotes the elementary charge (1.602 × 10^‐19^[C]). Figure 2b shows the calculated design value of *K(E)*. In the figure, 1 MU corresponds to 5 × 10^7^ particles in the case of a 430‐MeV/n carbon beam (range 30 cm) and half that number of particles for a 140‐MeV/n beam (range 4 cm). The rest of this paper expresses the number of carbon particles detected by the dose monitor in units of MU.

#### 2.A.2 IDD data

We used both dose measurement results and Monte Carlo simulation to model *IDD_n_(z)* (*n* = 1,2,3) data. The Geant4.9.3 ^10^ platform provided the Monte Carlo calculation code, and the irradiation apparatus shown in Figure 1 was implemented accordingly in the simulation. The IDD components of the incident ^12^C particles, *IDD_12C_(z)*, and of the fragment particles, *IDD_frag_(z)*, were then determined through both the dose measurements and the Monte Carlo simulation. The calculated results from the simulation were corrected to reproduce the measured IDD data from a large‐area ionization chamber, the Stingray (IBA Dosimetry), whose detection diameter is 12 cm. Because the total cross section in the Geant4 Monte Carlo code was assumed to differ from the true value, the calculated IDD did not agree with the Stingray measurement results, so we developed correction methods in terms of the total cross section.

First, we express *IDD_12C_*, ie the dose contribution from ^12^C, by multiplying the ^12^C fluence distribution *Φ(z)* at depth *z* and the convolution of the stopping power *S(r)* in terms of the range straggling *σ_strag_*:

(7) $$IDD_{12C}\left(z;R_{0},\lambda \right)=\Phi \left(z;\lambda \right)\times \int_{‐\infty }^{+\infty }dz^{\prime }\exp\left(‐\frac{{\left(z^{\prime }‐z\right)}^{2}}{2\sigma_{\mathrm{strag}}^{2}}\right)\cdot S\left(R_{0}‐z^{\prime }\right),$$

where *R_0_* denotes the initial residual range, *S(r)* is the stopping power of ^12^C for residual range *r*, and *λ* is a quantity related to the total cross section. The ^12^C particle fluence *Φ(z; λ)* is then reduced, according to the depth *z*, by nuclear reaction:

(8) $$\Phi \left(z;\lambda \right)=\Phi_{0}\exp\left(‐\frac{\sigma N_{A}\rho }{M}z\right)=\Phi_{0}2^{‐z/\lambda },$$

where *Φ_0_* denotes the initial particle fluence, *N_A_* is the Avogadro number, *ρ* is the density of the material, *M* is the molecular weight, and *σ* is the total cross section. In Eq. (8), the quantity *λ* is defined as

(9) $$\lambda =\frac{\ln2∙M}{\sigma N_{A}\rho },$$

meaning that *λ* is inversely proportional to *σ*. Note that, in the convolution calculation in Eq. (7), the stopping power *S(r)* is tabulated from an ICRU73 data table, and the straggling parameter *σ_strag_* is kept constant independently of the depth. By tuning *λ* and *σ_strag_*, we can use Eq. (7) to well reproduce the relative distribution of Geant4 *IDD_12C_(z)* scored results. Correction for *IDD_12C_(z)* only pertains to the fluence term *Φ(z; λ)*, and thus, the correction formula for *IDD_12C_(z)* is derived as

(10) $$\frac{IDD_{12C}\left(z;aftercorrection\right)}{IDD_{12C}\left(z;beforecorrection\right)}=\frac{\Phi \left(z,\lambda^{\prime }\right)}{\Phi \left(z,\lambda \right)}=\frac{2^{‐z/\lambda^{\prime }}}{2^{‐z/\lambda }},$$

where *λ* and *λ’* are adjustment parameters for *IDD_12C_(z)* before and after correction respectively. The fragment component *IDD_frag_(z)* is assumed to scale with the total cross section; that is, we assume a linear relationship for *IDD_frag_(z)* with the total cross section, giving the following correction formula:

(11) $$\frac{IDD_{\mathrm{frag}}\left(z;aftercorrection\right)}{IDD_{\mathrm{frag}}\left(z;beforecorrection\right)}=\frac{\sigma^{\prime }}{\sigma }=\frac{\lambda }{\lambda^{\prime }}.$$

**Fig. 3:**
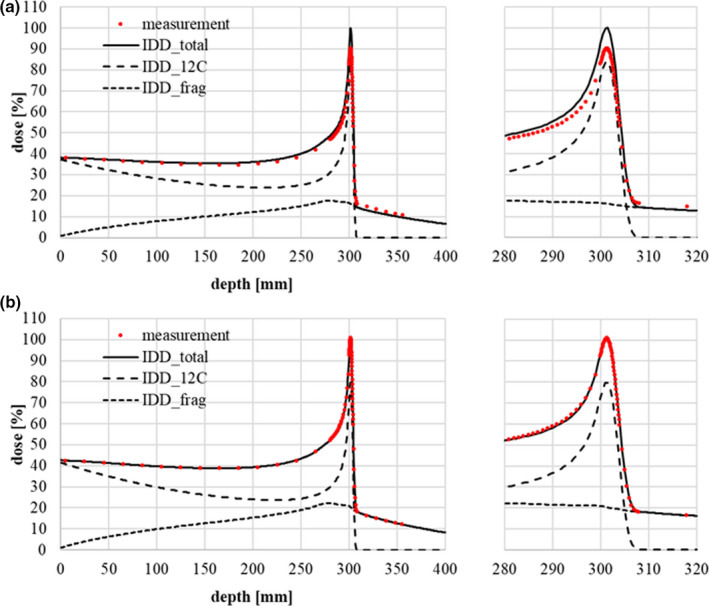
Relative IDD data for Geant4 calculations adjusted to fit measurements with a Stingray chamber (a) before correction and (b) after correction. The red points represent the dose measurements. The solid, dashed, and dotted lines represent *IDD_total_ (IDD_12C_ + IDD_frag_)*, IDD_12C_, and *IDD_frag_* respectively. The correction parameters in Eqs. (10) and (11) are *λ* = 170 mm (before correction) and *λ’*=150 mm (after correction).

Figure 3 shows the results of IDD correction for a 430‐MeV/n carbon beam obtained by the above methods, together with corrected Monte Carlo results reproducing the measured result within ±2% in the proximal and distal regions.

After adjusting the relative IDD shape to the dose measurements, the IDD data are converted to absolute dose values reflecting the absolute dose measurements at a shallow depth (2 cm), as described in 11. The dose area product (DAP) at a 2‐cm depth was measured by rectangular uniform grid irradiation using a mono‐energy beam ^12^. The DAP measurement was performed with an advanced Markus chamber, and the absolute dose was measured at the center of the field where lateral dose distribution is flat. The irradiation number of carbon particles is uniform, with a value of *Q* [MU] per spot, and the spot spacing Δ is 3 mm in both the x and y directions. Then, the DAP per MU is defined as

(12) $$DAP\left(z=2\;cm\right)=D_{\mathrm{meas}}\times \frac{\Delta^{2}}{Q},$$

where *D*_meas_ denotes the measured dose in units of Gy at the center of the field. We introduce the total IDD as

(13) $${\mathrm{IDD}}_{\mathrm{total}}\left(z\right)={\mathrm{IDD}}_{12C}\left(z\right)+{\mathrm{IDD}}_{\mathrm{frag}}\left(z\right).$$

Next, using the corrected relative *IDD_12C_(z)* and *IDD_frag_(z)* in Eqs. (10) and (11), the absolute IDD *IDD_total_^abs^(z)* can be obtained as.

(14) $${\mathrm{IDD}}_{\mathrm{total}}^{\mathrm{abs}}\left(z\right)=\frac{{\mathrm{IDD}}_{\mathrm{total}}\left(z\right)}{{\mathrm{IDD}}_{\mathrm{total}}\left(z=2\;cm\right)}\times DAP\left(z=2\;cm\right).$$

The dimensions of the *DAP* and *IDD* in Eqs. (12) and (14) are [Gy·mm^2^/MU]. The *IDD_12C_^abs^(z)* and *IDD_frag_^abs^(z)* of each scanning beam are determined by the above‐stated correction method in absolute dose units. The obtained *IDD_12C_^abs^(z)* is equal to the first component *IDD_1_(z)* of the triple Gaussian, while the fragment component *IDD_frag_^abs^(z)* corresponds to the sum of the second and third components, including the detection efficiency of the third component:

(15) $${\mathrm{IDD}}_{12C}^{\mathrm{abs}}\left(z\right)={\mathrm{IDD}}_{1}\left(z\right),$$

(16) $${\mathrm{IDD}}_{\mathrm{frag}}^{\mathrm{abs}}\left(z\right)={\mathrm{IDD}}_{2}\left(z\right)+{\varepsilon \times IDD}_{3}\left(z\right),$$

where *ε* denotes the detection efficiency of the Stingray chamber as explained later, in Section 2.A.4.

#### 2.A.3 Beam size of ^12^C

The first component in Eqs. (2) and (3) corresponds to the incident ^12^C particles. Its beam sizes *σ_1,x_(z), σ_1,y_(z)* are calculated by considering both the beam’s optical parameters and multiple Coulomb scattering. The beam transport equations including multiple scattering according to Fermi‐Eyges theory are.

(17) $$\sigma_{11}\left(z\right)=\sigma_{11}\left(z=0\right)+2\sigma_{12}\left(z=0\right)z+\left(\sigma_{22}\left(z=0\right)+\frac{\Delta \theta^{2}}{3}\right)z^{2},$$

(18) $$\sigma_{12}\left(z\right)=\sigma_{12}\left(z=0\right)+\left(\sigma_{22}\left(z=0\right)+\frac{\Delta \theta^{2}}{2}\right)z,$$

(19) $$\sigma_{22}\left(z\right)=\sigma_{22}\left(z=0\right)+\Delta \theta^{2},$$

where *σ_11_(z), σ_12_(z), σ_22_(z)* are the phase‐space parameters at depth *z*, *Δθ^2^* is the increment of the angular divergence when passing through distance *z*, and *z* = 0 denotes the initial point of transportation. The beam size is calculated by the square root of *σ_11_(z)* as

(20) $$\sigma_{1,x}\left(z\right)=\sqrt{\sigma_{11}\left(z\right)}.$$

Equations (17), (18), (19), and (20) are expressions in the x direction, and the same equations also hold in the y direction. The formula for the angular divergence increment was improved to a suitable form for treatment planning by Kanemastsu ^13^ :

(21) $$\Delta \theta^{2}=C\times Z^{‐0.16}\times A^{‐0.92}\times \ln\left(\frac{R}{R^{\prime }}\right),$$

where *R* and *R’* are the initial and residual ranges, respectively, *Z* is the atomic number, *A* is the mass number of the incident particles, and *C* is an adjustment parameter for the scattering power.

**Fig. 4:**
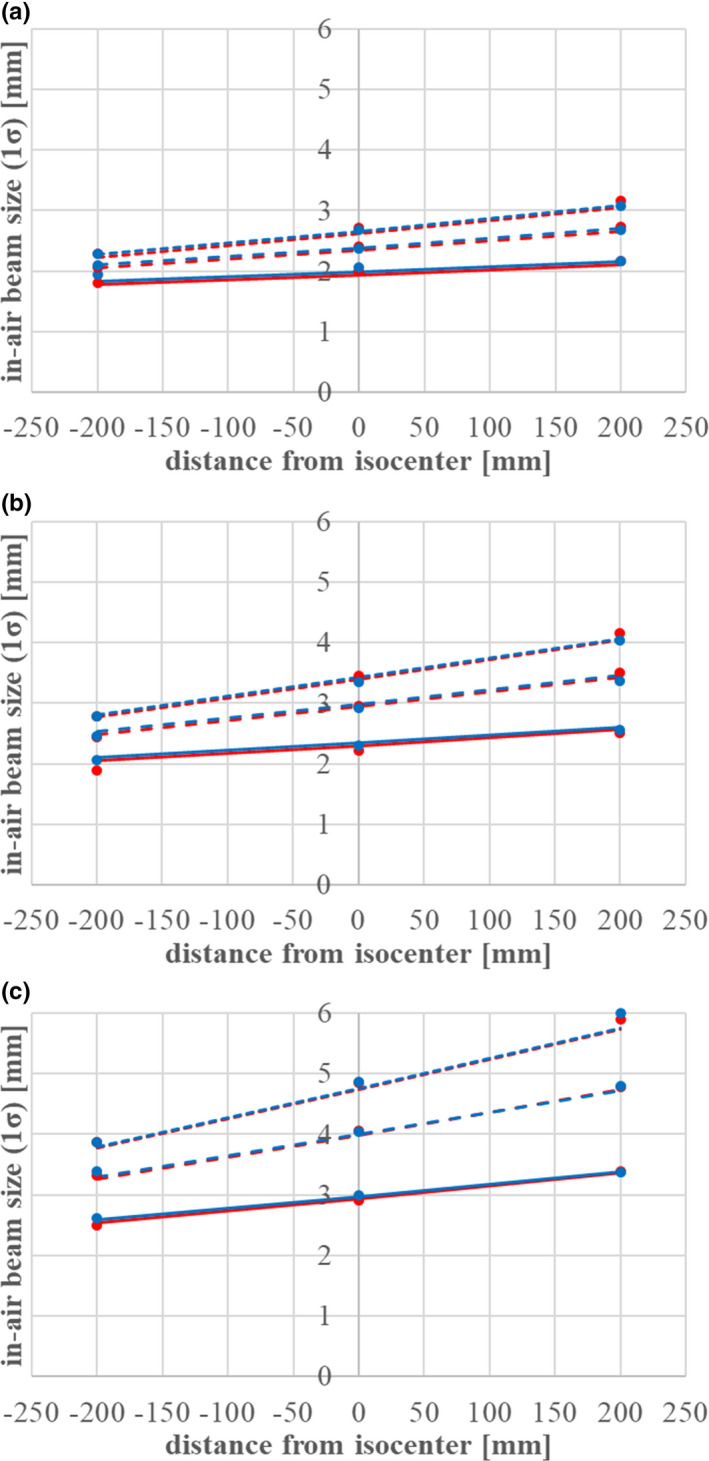
In‐air beam size measurement results with fluorescent screen monitors and calculation results from an analytical Moliere program for (a) 430 MeV/n, (b) 302.1 MeV/n, and (c) 208.3 MeV/n. The red and blue points represent the measurements in the x and y directions respectively. The solid, dashed, and dotted lines show the calculation results for RS=0 mm, RS=12 mm (medium thickness), and RS=24 mm (maximum thickness).

**Fig. 5:**
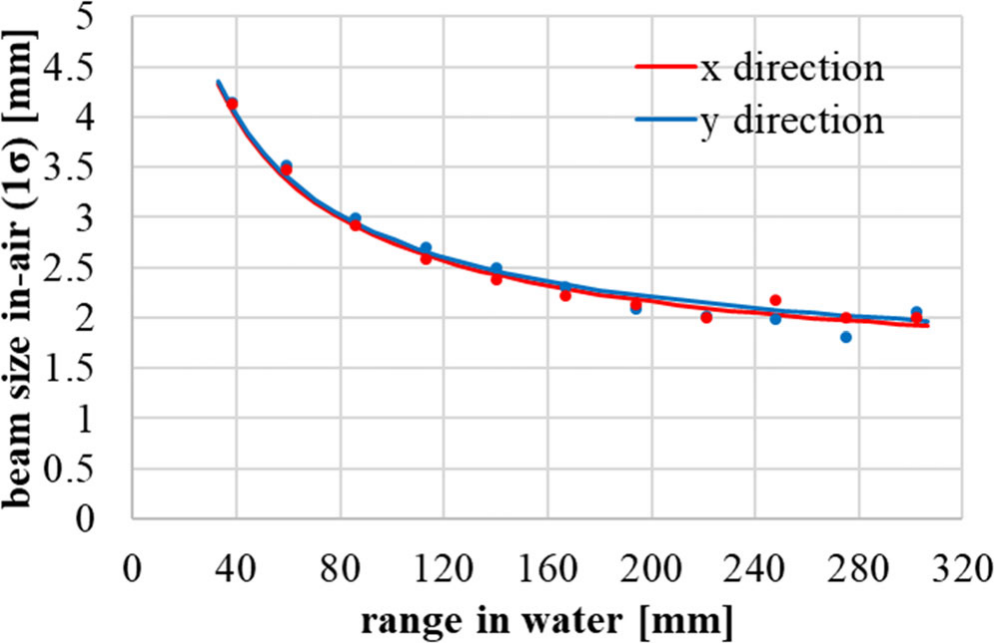
In‐air beam size (1σ) at the isocenter from 40 to 300 mm. The points represent the measurement results with fluorescent screen monitors, and the lines represent the calculation results. Red and blue components correspond to the x and y directions respectively.

The beam data for the beam size of the first component consists of *σ_11_(z), σ_12_(z), σ_22_(z)* at the isocenter in the x and y directions in Eqs. (17), (18), and (19) (six parameters to characterize the phase space in x and y) and the scattering power parameter *C* in Eq. (21). To determine these parameters, we performed in‐air dose measurement with fluorescent screen monitors and in‐water dose measurement of the lateral beam size with a pinpoint chamber. In‐air dose measurements were collected at three points just at the, upstream and downstream the isocenter (displacements of 0 mm, ±200 mm) to trace the beam size behavior. The beam size data measured by the fluorescent screens was fitted with a two‐dimensional single Gaussian and analyzed with the design values of the beam’s optical parameters and analytical code based on the Moliere theory of multiple Coulomb scattering. By tuning the design values and the scattering power in the code, we could obtain agreement between the calculated beam size and the measurement results. Figure 4 shows the in‐air beam size measurements at three positions in the cases of (a) 430 MeV/n, (b) 302.1 MeV/n, and (c) 208.3 MeV/n, with range‐shifter thicknesses of 0 mm, 12 mm (medium thickness), and 24 mm (maximum thickness). Figure 5 shows the beam size for 11 energies ranging from 140 to 430 MeV/n without range shifters. In addition, the parameter C was adjusted to 0.0015 to reflect the beam size measurements in water at three depths with three beam energies (140, 302, and 430 MeV/n).

The beam splitting algorithm in phase space is applied to the first component to appropriately reflect the lateral heterogeneity in a computed tomography (CT) image. The second and third components are calculated only considering the density information along the central axis of the scanning beam, because the beam sizes of the second and third components are large, while the dose contributions are small. From the viewpoints of dose calculation accuracy and speed, we chose a fixed number of split subbeams in phase space as described in Kanematsu ^14^.

#### 2.A.4 Determination of fragment components

**Fig. 6:**
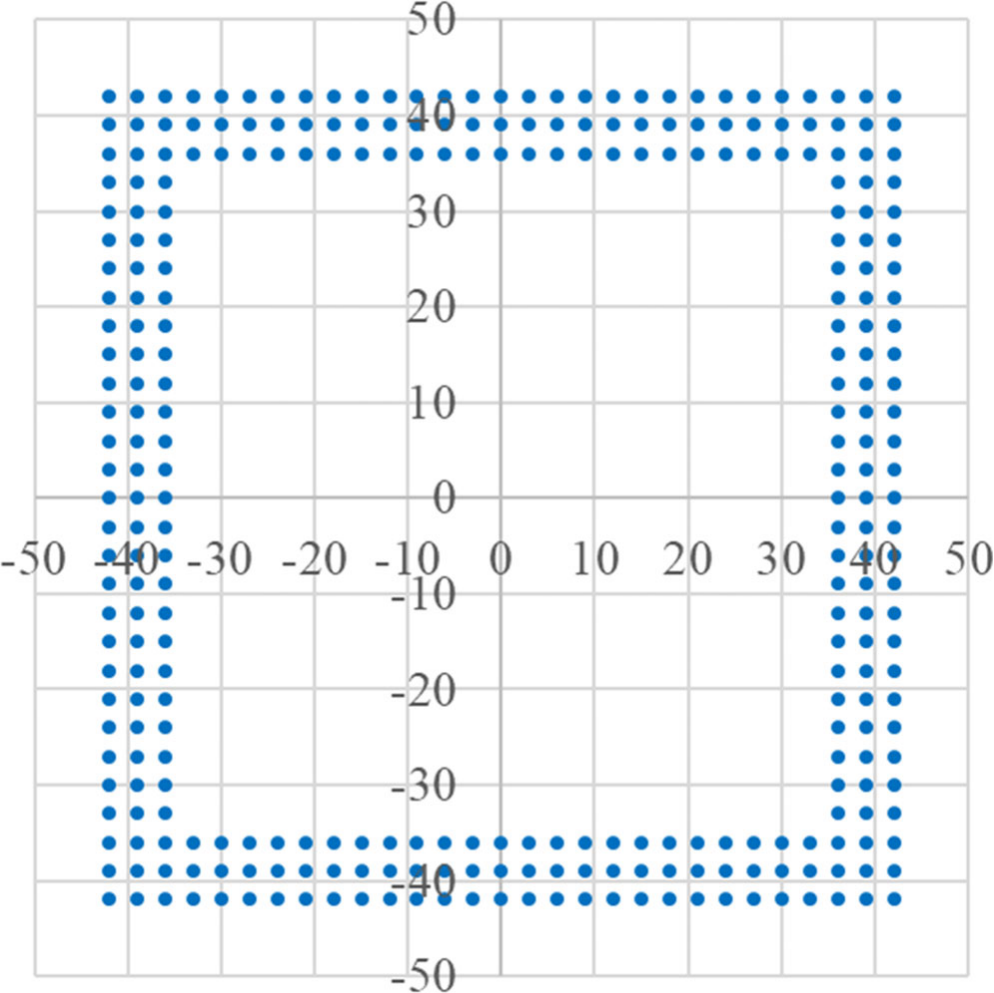
Spot arrangement for frame pattern irradiation, corresponding to pattern F in Table 1. The spot interval is 3 mm in the x and y directions. The inner and outer spot positions are ±36 mm and ±42 mm respectively.

The second and third components’ parameters, ie *IDD_2_(z), IDD_3_(z), σ_2_(z), σ_3_(z)*, in Eqs. (2) and (4) have to be determined to describe the distant fragment halo component. There are two methods of dose measurement to determine the distant component of a pencil beam: one is field size factor (FSF) measurement, as described by Zhu ^11^ using various sizes of square fields, and the other is frame pattern irradiation, as described by Inaniwa ^15^. In both cases, the absolute dose at the center of the field is measured with a pinpoint chamber. We chose the frame pattern irradiation method, because the primary component with a large dose contribution is absent in the detection area, so we expected to be able to model the second and third components more accurately. We exploited the seven irradiation frame patterns listed from A to G in Table 1. Figure 6 shows the spot configuration of frame pattern F. Absolute dose measurements were performed at the center of the field with the pinpoint chamber, by combining various beam energies and range‐shifter thicknesses as listed in Table 2. The IDD and beam size of the first component had already been obtained, as described in Section 2.A.3, so parameter fitting of *IDD_2_(z), IDD_3_(z), σ_2_(z), σ_3_(z)* was performed using Eq. (1) by setting *i* as the origin, ie the center of the field, and summing the dose contributions from beam *j* according to the frame pattern configurations in Table 1.

**Table 1:** Details of spot configurations for frame pattern irradiation.

Pattern	Inner spot position [mm]	Outer spot position [mm]	Number of spots
A	0	12	81
B	12	18	120
C	18	24	168
D	24	30	216
E	30	36	264
F	36	42	312
G	42	48	360

**Table 2:** Details of frame pattern measurements for various combinations of the beam energy, range‐shifter thickness, and irradiation pattern.

No.	Energy [MeV]	Range shifter [mm]	No.	Energy [MeV]	Range shifter [mm]
1	430	0	13	302.1	3
2	430	3	14	302.1	12
3	430	12	15	302.1	24
4	430	24	16	272.8	0
5	406.2	0	17	241.9	0
6	381.6	0	18	208.3	0
7	381.6	3	19	170.9	0
8	381.6	12	20	170.9	3
9	381.6	24	21	170.9	12
10	356.1	0	22	170.9	18
11	329.6	0	23	140	1.4
12	302.1	0			

**Fig. 7:**
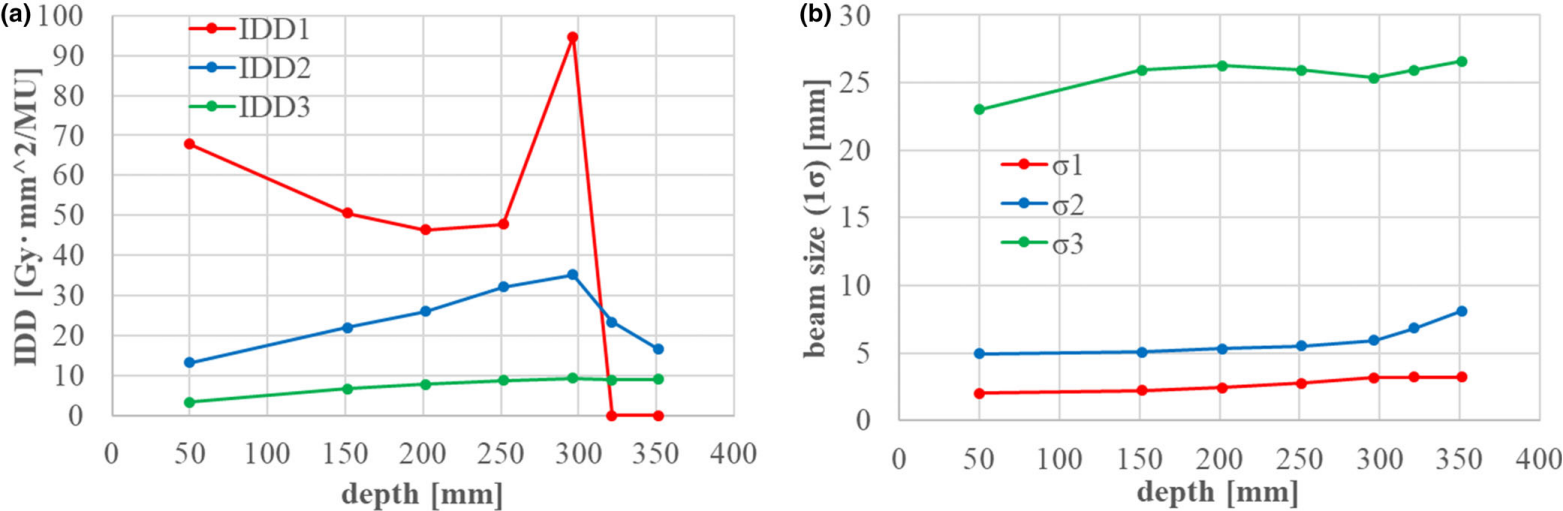
Fitting results for (a) IDD and (b) the beam sizes of the second and third components. IDD2, IDD3, σ2, and σ3 were fitted as free parameters. IDD1 and σ1 were already obtained as in sections 2.1.2 and 2.1.3.

Figure 7 shows the fit results for the IDD and beam size in the case of 430 MeV/n. For each frame pattern, dose measurements were performed at seven depths. In the figure, the beam size of the third component is almost constant, and we observed a similar tendency at other energies. Considering these results and previous research in 7, we fix the beam size of the third component as 25 mm (1σ). On the other hand, the beam size of the second component, *σ_2_(z)*, and the IDD of the third component, *IDD_3_(z)*, change gradually with depth, so we express these parameters as analytical functions. *IDD_2_(z)* is determined by subtracting *IDD_3_(z)* from *IDD_frag_(z)* and considering the detection efficiency of the third component in Eq. (16), determined as

(22) $$\varepsilon =\int_{0}^{2\pi }d\theta \int_{0}^{R}\frac{1}{2\pi \sigma_{3}^{2}}\exp\left(‐\frac{r^{2}}{2\sigma_{3}^{2}}\right)dr,$$

where *R* denotes the radius of the Stingray chamber’s detection area (6 cm). The efficiency of the third component *ε* is calculated as 0.944 for *R* = 6 cm and σ*_3_* = 25 mm, while the efficiency of the first and second components is 1, meaning full collection by the chamber.

​

**Fig. 8:**
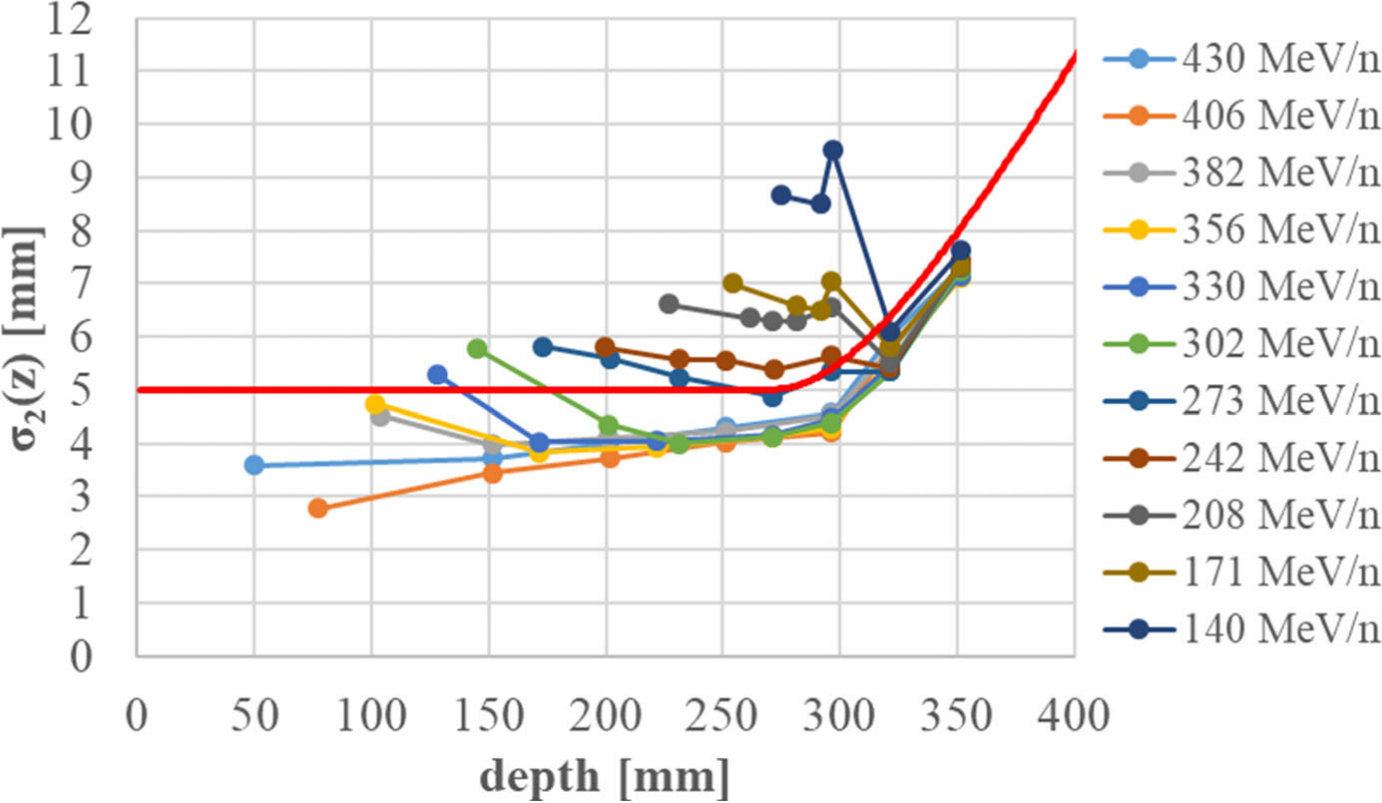
Fitting results for the beam size of the second component *σ_2_(z)* without range shifters. The Bragg‐peak depths from 140 to 406 MeV/n are shifted to that for 430 MeV/n. The red line shows the analytical function describing *σ_2_(z)*.

**Fig. 9:**
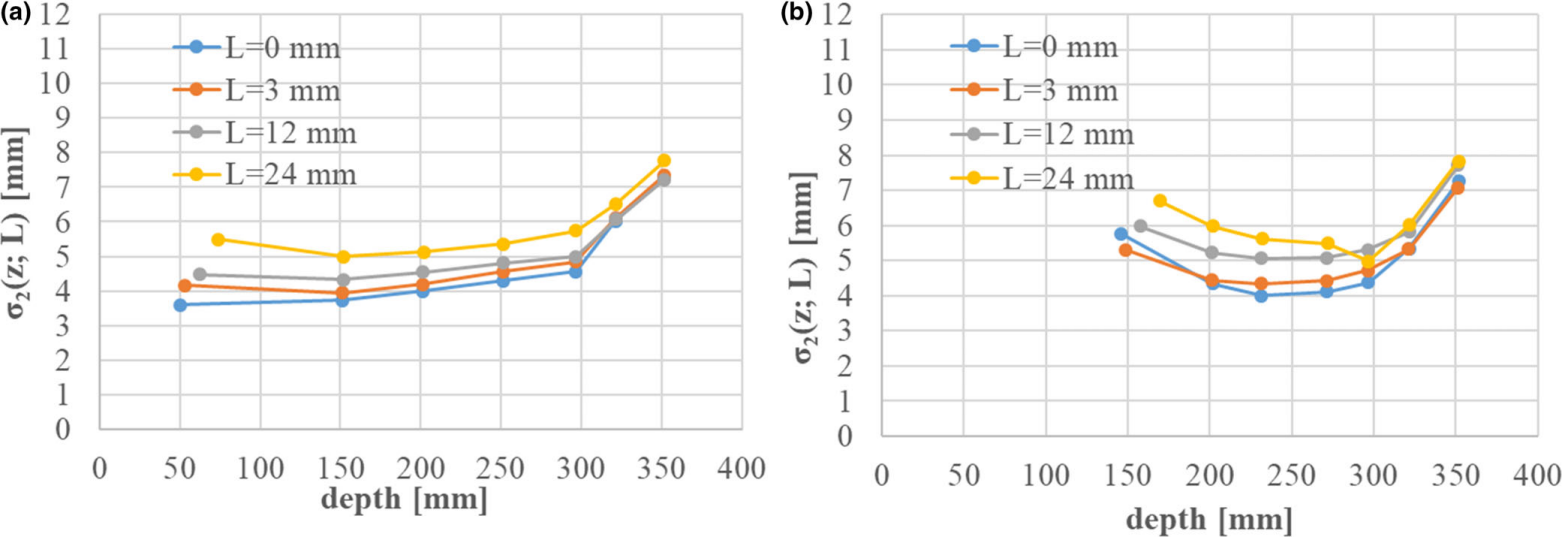
Fitting results for the beam size of the second component, *σ_2_(z; L)*, with range‐shifter thickness *L* [mm] in the cases of (a) 430 MeV/n and (b) 302.1 MeV/n. The Bragg‐peak depths are shifted to that for 430 MeV/n.

We again fitted parameters *IDD_2_(z), IDD_3_(z),* σ*_2_(z)* with the condition of σ_3_ = 25 mm. Figure 8 shows the fitted results for σ*_2_(z)* with various energies (Table 2) without range shifters. In the figure, all data are shifted along the horizontal axis by adjusting the Bragg peak position of each beam to that of the 430‐MeV/n beam. We expressed σ*_2_(z)* without range shifters by using a hyperbolic function form:

(23) $$\sigma_{2}\left(z\right)=\left\{\begin{array}{cc} C_{1} & z\leq z_{p}‐C_{2}\\ \sqrt{C_{1}^{2}+C_{3}{\left(z‐z_{p}+C_{2}\right)}^{2}} & z\geq z_{p}‐C_{2} \end{array}\right.,$$

where *z_p_* represents the carbon Bragg‐peak depth, and the parameter values are *C_1_* = 5 [mm], *C_2_* = 30 [mm], *C_3_* = 0.006. Next, Figure 9 shows the fitted results for σ*_2_(z; L)* when inserting range shifters with water equivalent thickness *L* [mm] for (a) 430 MeV/n and (b) 302 MeV/n. In the figure, σ*_2_(z; L)* gets larger as the range shifter gets thicker. In modeling the beam size of the second component with range shifters inserted, we assume that the resulting increase in the beam size of the second component is equal to that of the first component. The beam size of the second component, σ*_2_(z; L)*, with range shifters of water equivalent thickness *L*, is calculated by

(24) $$\sigma_{2}\left(z;L\right)=\sqrt{{\sigma_{2}\left(z+L\right)}^{2}+{\sigma_{1}\left(z;L\right)}^{2}‐{\sigma_{1}\left(z+L\right)}^{2}},$$

where *σ_1_(z; L)* is the beam size of the first component with range shifters, and σ*_1_(z + L)* and σ*_2_(z + L)* are the respective beam sizes of the first and second components at depth *z + L* without range shifters. In Eq. (24), σ*_1_(z*; *L)^2^* ‐ σ*_1_(z + L)^2^* corresponds to the squared beam size difference in the first component with and without range shifters. It is calculated from the modeled results for the first component, as described in Section 2.A.3.

**Fig. 10:**
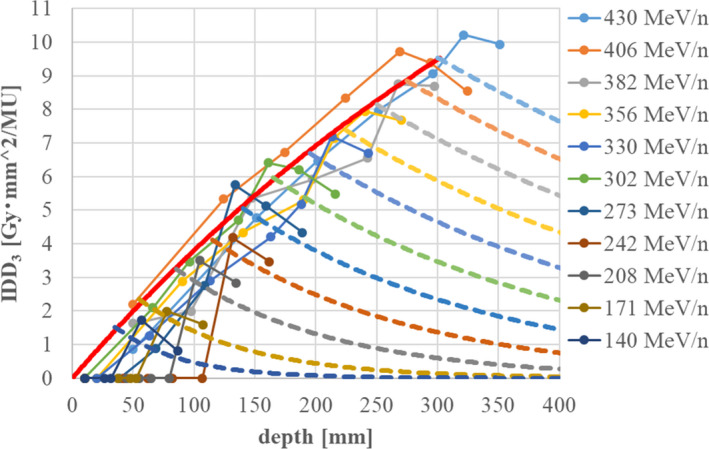
Fitting results for the IDD of the third component, *IDD_3_(z)*. The red line shows the increasing component up to the Bragg‐peak depth, while the dashed lines show the decreasing components exceeding that depth.

Figure 10 shows the fitted results for *IDD_3_(z)*. As seen here, *IDD_3_(z)* increases up to the Bragg‐peak depth and then decreases at depths exceeding the Bragg peak. This tendency holds regardless of the incident energy. Therefore, we approximate *IDD_3_(z)* in the same exponential function form as

(25) $$IDD_{3}\left(z\right)=\left\{\begin{array}{cc} C_{4}\left[1‐\exp\left(‐\frac{z}{C_{5}}\right)\right] & z\leq z_{p}\\ C_{4}\left[1‐\exp\left(‐\frac{z_{p}}{C_{5}}\right)\right]\exp\left(‐\frac{z‐z_{p}}{C_{6}z_{p}}\right) & z\geq z_{p} \end{array},\right.$$

where *z_p_* denotes the Bragg‐peak depth, and the parameters are *C_4_* = 21 [Gy･mm^2^/MU], *C_5_* = 500 [mm], and *C_6_* = 1.5. With the range shifters inserted, *IDD_3_(z)* is shifted in the depth direction according to

(26) $${\mathrm{IDD}}_{3}\left(z;L\right)={\mathrm{IDD}}_{3}\left(z+L\right),$$

where *IDD_3_* *(z; L)* denotes the third component with range‐shifter of water equivalent thickness L.

#### 2.A.5 Determination of correction factor (IDNF)

Here, we define the integral dose normalization factor (IDNF), which is a correction factor for each IDD to compensate for the difference from the measured absolute dose. Our IDNF factor works like the depth‐dose normalization factor (DDNT) in 11. We determine the IDNF by comparing the absolute calculated dose value with absolute dose measurement results for several volume irradiations. We introduce this factor to correct the ambiguity of the absolute dose for the DAP at a 2‐cm depth. Table 3 summarizes the conditions of volume irradiation used to determine the IDNF.

**Table 3:** Conditions of volume irradiation for IDNF correction.

Range [cm]	SOBP width [cm]	Field size [cm]
30	10	2,4,6,8,10,15,20
20	8	2,4,6,8,10,15,20
12	4	2,4,6,8,10,15,20
8	4	2,4,6,8,10,15,20
2	1	2,4,6,8,10,15,20

### 2.B Biological dose modeling

#### 2.B.1 Biological model

A biological model for carbon therapy was developed by NIRS to start passive irradiation treatment.^4^, ^5^, ^6^ Cell survival is calculated by an LQ model:

(27) $$S_{i}=\exp\left(‐\alpha_{i}d_{i}‐\beta_{i}{d_{i}}^{2}\right),$$

where *i* denotes the calculation point or voxel, *S_i_* is the cell survival, *d_i_* is the physical dose, and α*_i_* and β*_i_* are the LQ parameters. The physical dose *d_i_* is calculated by summing over the contributions of all scanning beams via Eq. (1). In the case of mixed irradiation from various scanning beams, *α_i_* and *β_i_* are each calculated by a dose‐averaged sum of each beam (*j*) contribution:

(28) $$\alpha_{i}=\frac{1}{d_{i}}\sum_{j}\alpha_{\mathrm{ij}}d_{\mathrm{ij}}w_{j}\;\;\;\;\sqrt{\beta_{i}}=\frac{1}{d_{i}}\sum_{j}\sqrt{\beta_{\mathrm{ij}}}d_{\mathrm{ij}}w_{j},$$

where *α_ij_* and *β_ij_* are the LQ parameter contributions from beam j to point *i*. The physical dose contribution *d_ij_* has a Gaussian distribution, as in Eq. (2), in the lateral direction, while the LQ parameters α*_ij_* and β*_ij_* are assumed to have no lateral distribution and are only functions of the LET value at the specified depth. Next, we introduce a biological effect at point *i* as

(29) $$e_{i}=\alpha_{i}d_{i}+\beta_{i}{d_{i}}^{2}.$$

Then, the number of particles of beam *j*, *w_j_*, is optimized to make the biological effect *e_i_* match a goal value *E_i_*. An evaluation function is calculated by summing the squares of the residuals from the goal value over all points:

(30) $$F\left(w_{j}\right)=\sum_{i}{\left(e_{i}‐E_{i}\right)}^{2},$$

where the optimization uses a quasi‐Newton algorithm with boundaries (L‐BFGS‐B). The biological effect goal value *E_i_* is fixed as the HSG cell 10% survival, giving *E_i_ = ‐ln(0.1)≃2.3*. After determining the particle number *w_j_* of each beam, the physical dose *d_i_* and LQ parameters *α_i_* and *β_i_* are calculated according to Eqs. (1) and (28), and then the biological effect *e_i_* is calculated by Eq. (29).

Next, the photon equivalent dose, ie the biological dose *d_bio,i_*, which is the physical dose of standard radiation (ie a 200‐keV X‐ray) to induce the same effect, is calculated using the LQ parameters of a photon (α*_X_* = 0.33 [Gy^‐1^], β*_X_* = 0.06 [Gy^‐2^]) via Eq. (31) below. By considering the clinical results of a neutron therapy experiment, the biological dose is multiplied by 1.46 to obtain the clinical dose, as in Eq. (32):^4^, ^5^

(31) $$d_{bio,i}=\frac{\sqrt{{\alpha_{X}}^{2}+4\beta_{X}e_{i}}‐\alpha_{X}}{2\beta_{X}},$$

(32) $$d_{clin,i}=1.46\times d_{bio,i}.$$

The clinical RBE is thus defined as the ratio of the clinical and physical doses:

(33) $${\mathrm{RBE}}_{clin,i}=\frac{d_{clin,i}}{d_{phys,i}}.$$

**Fig. 11:**
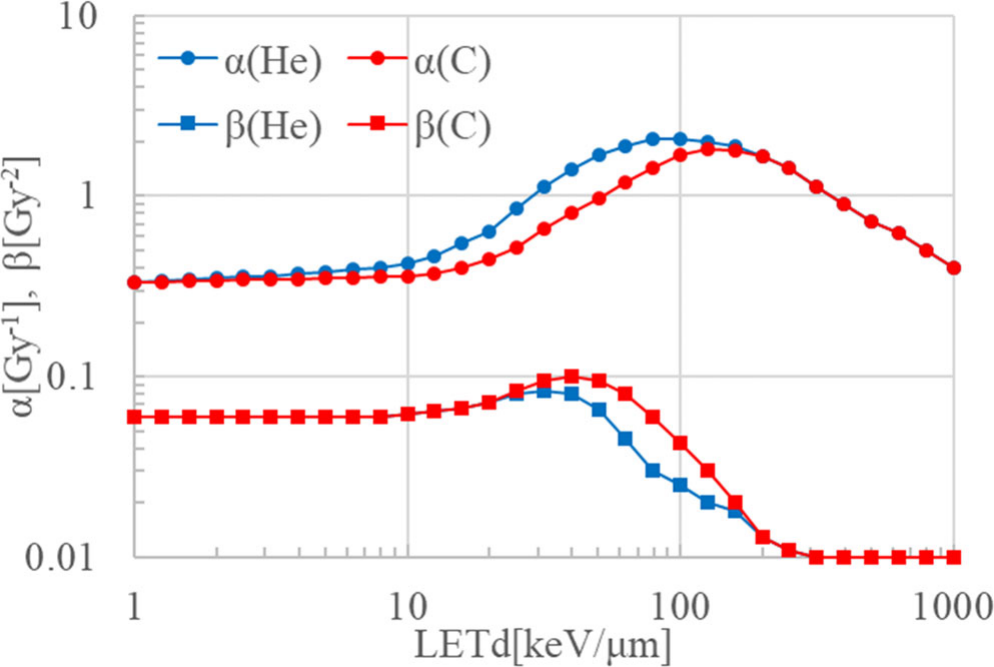
LQ parameters for carbon and helium, shown in red and blue respectively.

The above description is the established theory for passive irradiation since the start of carbon therapy in Japan. Recently, this model has been improved to use two kinds of LQ parameters, one for carbon and the other for helium.^16^ This improved method was developed for ridge‐filter design for passive irradiation, so we extended it to carbon beam scanning irradiation as follows. In the improved biological model described in 16, carbon LQ parameters α*^(C)^*, β*^(C)^* are applied for carbon isotopes, while helium LQ parameters α*^(He)^*, β*^(He)^* are applied for fragment isotopes other than carbon:

(34) $$\alpha_{i}=\frac{1}{d_{i}}\sum_{j}\left({\alpha_{\mathrm{ij}}}^{\left(C\right)}\left(z\right){d_{\mathrm{ij}}}^{\left(C\right)}+{\alpha_{\mathrm{ij}}}^{\left(He\right)}{{({\mathrm{LET}}_{\mathrm{frag}})d}_{\mathrm{ij}}}^{\left(frag\right)}\right)w_{j},$$

(35) $$\sqrt{\beta_{i}}=\frac{1}{d_{i}}\sum_{j}\left(\sqrt{{\beta_{\mathrm{ij}}}^{\left(C\right)}\left({\mathrm{LET}}_{C}\right)}{d_{\mathrm{ij}}}^{\left(C\right)}+\sqrt{{\beta_{\mathrm{ij}}}^{\left(He\right)}\left({\mathrm{LET}}_{\mathrm{frag}}\right)}{d_{\mathrm{ij}}}^{\left(frag\right)}\right)w_{j},$$

where *d_ij_^(c)^* and *d_ij_^(frag)^* are the physical dose contributions of the carbon isotopes and fragment isotopes respectively. Figure 11 shows the carbon and helium LQ parameters α*^(C)^*, β*^(C)^*_,_ α*^(He)^*, β*^(He)^* used in this study, with the carbon LQ parameters α*^(C)^* and β*^(C)^* in red and the helium LQ parameters α*^(He)^* and β*^(He)^* in blue. These LQ parameters are functions of the LET, so we prepared dose‐averaged LET (LETd) data for each scanning beam from the Geant4 calculation results.

#### 2.B.2 Beam data for biological dose

**Fig. 12:**
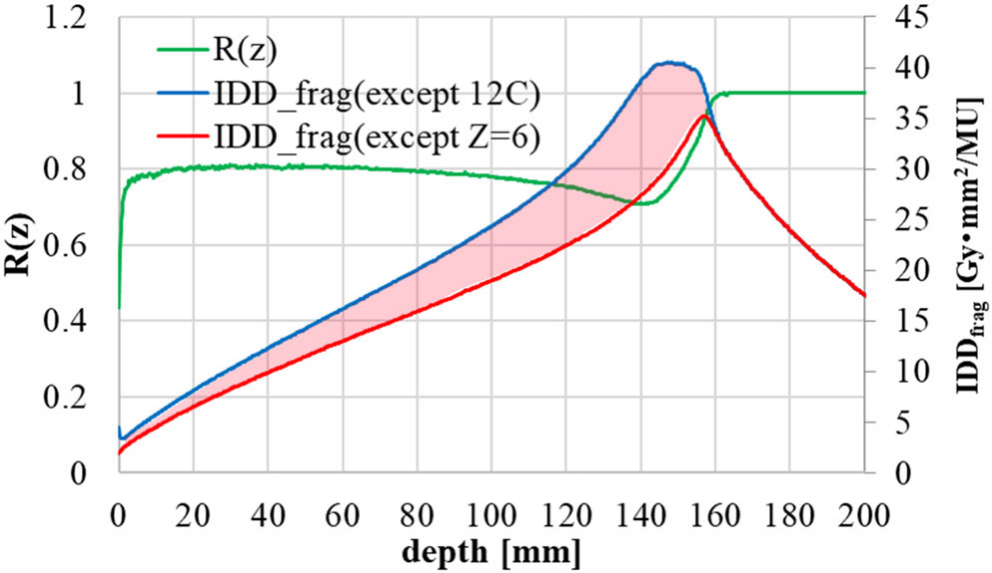
Ratio of *IDD*_*frag*_ without carbon isotopes (red line) to *IDD*_*frag*_ with carbon fragment isotopes (blue line), shown by the green line, in the case of 302.1 MeV/n.

The physical dose contributions of the carbon and fragment isotopes, *d_ij_^(C)^, d_ij_^(frag)^* in Eqs. (34) and (35), are related to the triple Gaussian components in the following way:

(36) $${d_{\mathrm{ij}}}^{\left(C\right)}={d_{\mathrm{ij}}}^{\left(1\right)}+\left(1‐R\left(z\right)\right){\mathrm{IDD}}_{\mathrm{frag}}\left(z\right)\times G_{2}\left(z\right),$$

(37) $${d_{\mathrm{ij}}}^{\left(frag\right)}=\left[R\left(z\right)\times {\mathrm{IDD}}_{\mathrm{frag}}\left(z\right)‐{\mathrm{IDD}}_{3}\left(z\right)\right]\times G_{2}\left(z\right)+{d_{\mathrm{ij}}}^{\left(3\right)},$$

where *IDD*_frag_ *(z)* denotes the sum of *IDD_2_(z)* and *IDD_3_(z)*, *G_2_(z)* is the lateral Gaussian distribution of the second component as in Eq. (4), and *R(z)* is the ratio of the *IDD_frag_* values including and excluding carbon isotopes. Here, we introduce *R(z)* because the second component of the triple Gaussian model is supposed to be a mixture of carbon isotopes such as ^11^C and other fragment isotopes from Z = 1 to Z = 5. This ratio *R(z)* is calculated by Geant4 as

(38) $$R\left(z\right)=\frac{{\mathrm{IDD}}_{\mathrm{frag}}\left(z;scored\;except\;Z=6\right)}{{\mathrm{IDD}}_{\mathrm{frag}}\left(z;scored\;except\;12C\right)},$$

where the *IDD_frag_(z; scored except Z = 6)* are scored without Z = 6 isotopes, and the *IDD_frag_(z; scored except ^12^C)* are scored without incident ^12^C particles. The numerator *IDD_frag_(z; scored except Z = 6)* thus excludes the effect of carbon fragment isotopes such as ^11^C, while the denominator *IDD_frag_(z; scored except ^12^C)* includes such effects. Therefore, *R(z)×IDD_frag_(z)* corresponds to the fragment contribution from Z = 1 to Z = 5, while *(1‐R(z))×IDD_frag_(z)* corresponds to the component for carbon fragment isotopes such as ^11^C. Table 4 summarizes the relationship between the triple Gaussian model and the applied LQ parameters. Figure 12 shows the Geant4 calculation results for the ratio of *IDD*_*frag*_ without carbon isotopes (red line) to *IDD*_*frag*_ with carbon fragment isotopes such as ^11^C (blue line) in the case of 302.1 MeV/n. The difference region (red shaded area) in the figure thus indicates the dose contribution of carbon fragment isotopes such as ^11^C. These *R(z)* data are calculated for each scanning beam and registered as beam data.

**Table 4:** Each component of the triple Gaussian model and the applied LQ parameters.

Triple Gaussian component	Nuclides	Beam size	LETd	Applied LQ parameters
1^st^	^12^C	σ_1,x_,σ_1,y_	Z = 6 (LET_C_)	carbon (α^(C)^, β^(C)^)
2^nd^	carbon isotopes (^11^C,etc.)	σ_2_(z)		
	heavier isotopes (Z = 3,4,5)		other than Z = 6 (LET_frag_)	helium (α^(He)^, β^(He)^)
3^rd^	lighter isotopes (Z = 1,2)	σ_3_(z)		

**Fig. 13:**
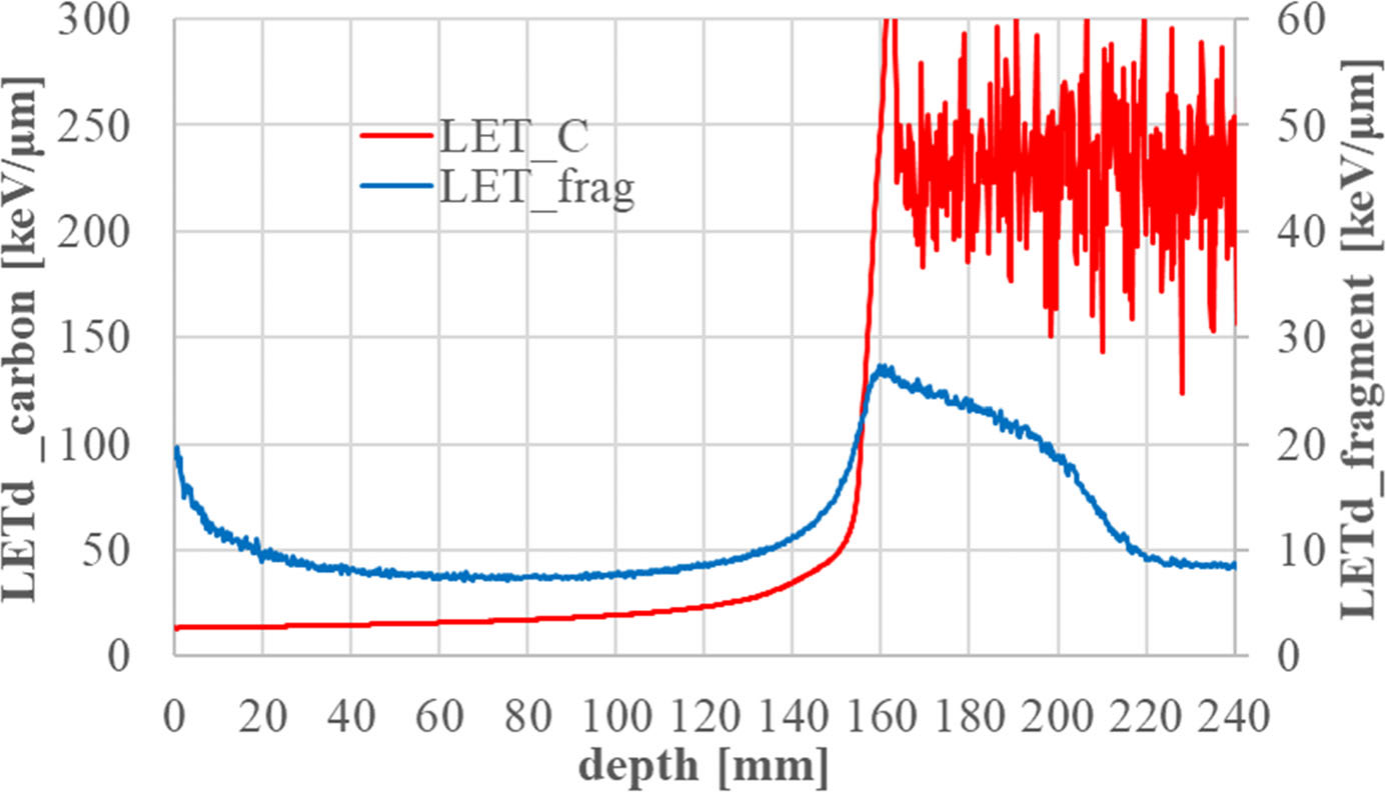
Dose‐averaged LET for carbon and fragment isotopes in the case of 302.1 MeV/n.

Next, the LETd data for obtaining the LQ parameters is also prepared by Geant4 according to

(39) $${\mathrm{LET}}_{d}\left(z\right)=\frac{\sum_{k}S_{k}\left(E_{k}\right)\times d_{k}}{\sum_{k}d_{k}}=\frac{\sum_{k}S_{k}\left(E_{k}\right)\times {\Delta E}_{k}}{\sum_{k}{\Delta E}_{k}},$$

where *k* denotes the *k*‐th event in Geant4, *S(E)* is the stopping power, and *d_k_* and *ΔE_k_* are the dose and energy deposit respectively. We use MSTAR ^17^ results for the stopping power *S(E)*. Event *k* in Eq. (39) concerns the case of Z = 6 particles for scoring the carbon LET (*LET_c_*) and cases other than Z = 6 particles for scoring the fragment LET (*LET_frag_*). Figure 13 shows dose‐averaged LET (*LET_d_*) data for carbon and fragment isotopes in the case of 302.1 MeV/n.

**Fig. 14:**
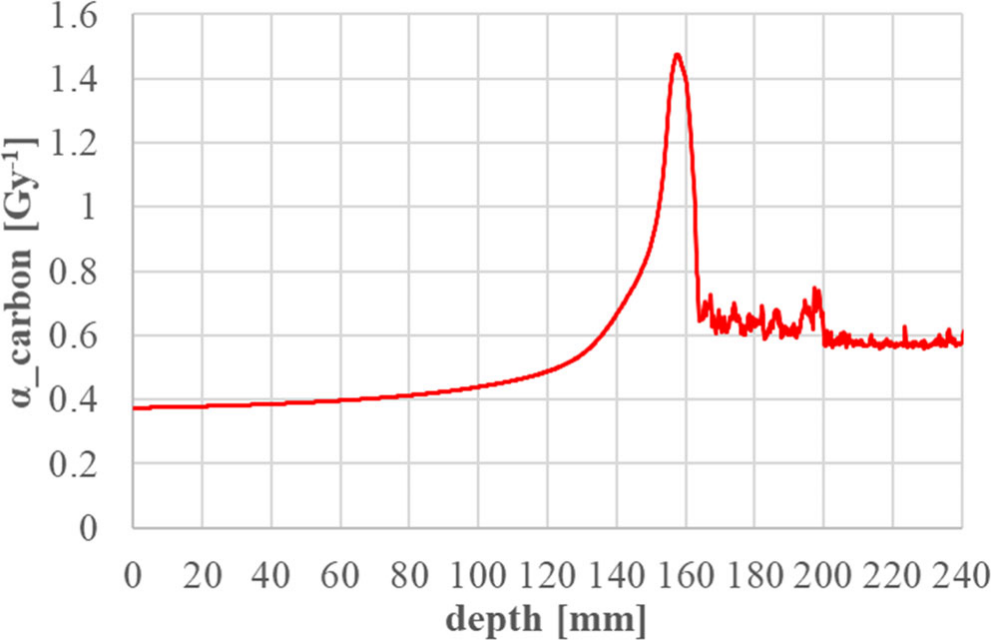
Dose‐averaged LQ parameter α for carbon isotopes in the case of 302.1 MeV/n.

Finally, the LQ parameters β*^(C)^*, α*^(He)^*, β*^(He)^* in Eqs. (34) (35) are calculated using the LQ table in Figure 11 via the dose‐averaged LET in Eq. (39). In addition, the LQ parameter α for carbon α*^(C)^* in Eq. (34) is directly calculated by Geant4 to consider a broad LET spectrum around the Bragg‐peak region as explained in Inaniwa ^3^ :

(40) $$\alpha^{\left(C\right)}=\frac{\sum_{k}\alpha \left(S_{k}\left(E_{k}\right)\right)\times d_{k}}{\sum_{k}d_{k}}=\frac{\sum_{k}\alpha \left(S_{k}\left(E_{k}\right)\right)\times {\Delta E}_{k}}{\sum_{k}{\Delta E}_{k}},$$

where event *k* is scored involving only Z = 6 particles. The dose‐averaged *α^(C)^* for carbon is directly scored and prepared as a function of depth *z*, as shown in Figure 14.

### 2.C Cell experiments

To verify the calculation accuracy of the physical and biological doses described above, we performed cell survival measurements under a biologically optimized spread‐out Bragg peak (SOBP) condition. The methods of these cell experiments were as follows.

#### 2.C.1 Cell line

A human salivary gland (HSGc‐C5) cell line was provided by the Japanese Collection of Research Bioresources Cell Bank. This cell line is known to have possibly been contaminated with HeLa cells. It is commonly used for in vitro models to study radiation therapy,^18^ however, so we used HSGc‐C5 cells in this study. These cells were maintained in a DMEM medium supplemented with 10% fetal bovine serum, 1% penicillin, streptomycin, and L‐glutamine (Thermo Fisher Scientific, Massachusetts, United States of America) at 37°C in a humidified atmosphere of 5% CO_2_, at less than 90% confluence.

#### 2.C.2 Colony formation assay

The HSGc‐C5 cells were seeded into 25‐cm^2^ plastic flasks and irradiated with carbon beam. After irradiation, the cells were washed in phosphate‐buffered saline (PBS), trypsinized, and seeded into dishes with a diameter of 60 mm. Two weeks after culturing, the cells were fixed with formalin and stained with crystal violet solution. After staining, colonies consisting of more than 50 cells were scored as survivors, and the survival fraction (SF) were calculated. All survival curves were fitted to the linear‐quadratic (LQ) model expressed in Eq. (27).

## 3 RESULTS

The appendix summarizes the beam data, such as the IDD, LET, and beam size, described in Section 2. The dose calculation accuracy was verified by comparing the results of dose measurement and past publication results described in 16.

### 3.A Physical dose

**Fig. 15:**
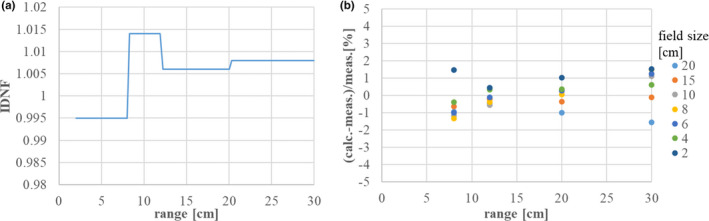
(a) IDNF for each scanning beam, and (b) differences between the absolute calculated physical dose and the dose measurement.

The calculated absolute physical dose was compared to absolute dose measurements after tuning the IDNF parameters. An SOBP was created by applying treatment planning software with the registered beam data, and dose measurements were performed with a Markus ionization chamber at the center of the SOBP. Figure 15 shows the results of IDNF correction and the final dose calculation accuracy. In the figure, the horizontal axis represents the range in water, while the vertical axis represents (a) the IDNF value and (b) the difference between the absolute calculated and measured doses. The final dose calculation accuracy was verified under the irradiation conditions listed in Table 3, and Figure 15b shows the field size dependency as a function of depth. The IDNF correction is within ±1.5%, and the final dose calculation accuracy is within ±2% in the range of 4 to 30 cm. We will report verification results in case of heterogeneity in a later submission.^19^

### 3.B Biological dose

**Fig. 16:**
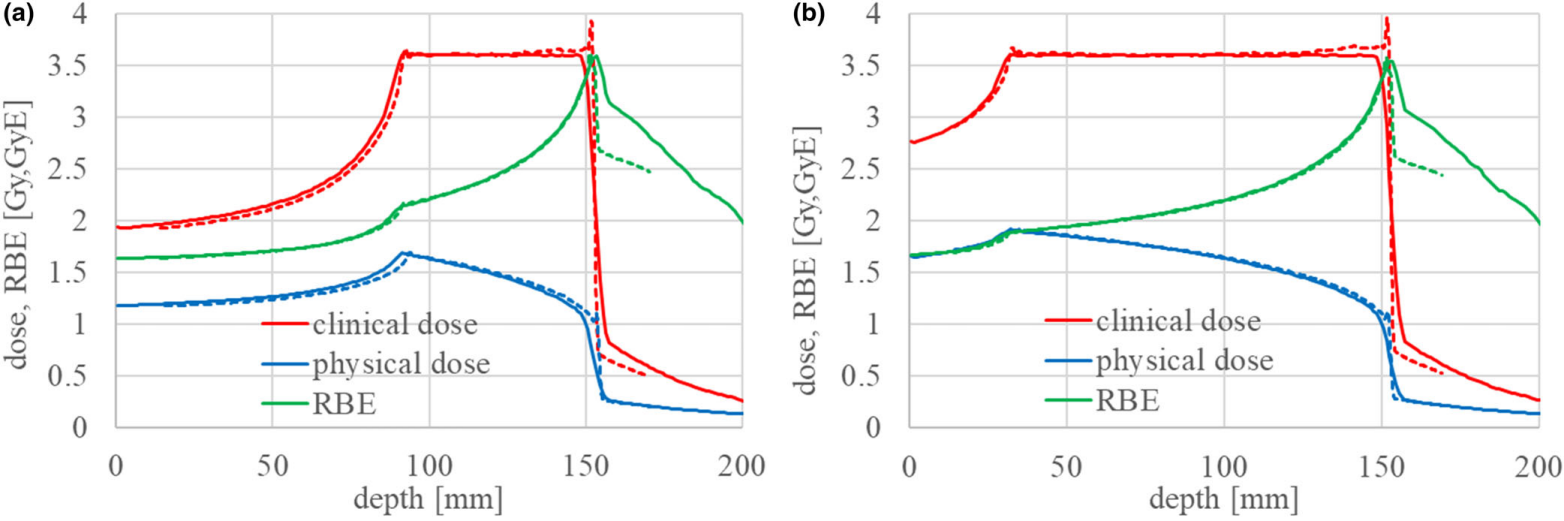
Clinical and physical dose distributions and RBE with an SOBP of (a) 6 cm and (b) 12 cm, for a range of 15 cm.

Figure 16 shows a comparison of the clinical and physical dose distributions and RBE to the passive irradiation results in 16. The figure shows the clinical dose, physical dose, and clinical RBE by solid red, blue, and green lines, respectively, while it shows the passive dose distributions by dotted lines of the same colors. The prescribed clinical dose was 3.6 GyE, and the range of the SOBP was about 15 cm in depth with a width of (a) 6 cm and (b) 12 cm. The dose distribution in this study well‐reproduced that of passive irradiation, except in the tail region beyond the SOBP’s flat region. Table 5 summarizes the clinical RBE results at the center of the SOBP with a range of 15 cm. The RBE value at the center agreed within ±1.5% of the passive results. An RBE discrepancy is observed in the tail region beyond the SOBP’s flat region, but physical dose distribution in this tail region is in good agreement. This was due to the calculation method for the biological effect of fragment particles. The calculation results of this study overestimate the clinical dose in the tail region by about 20% as compared to past dose distribution results.

**Table 5:** Comparison of clinical RBE at the center of the SOBP for a range of 15 cm.

SOBP width [cm]	This study	Gunma Univ. data
3	2.73	2.77
6	2.43	2.44
9	2.25	2.25
12	2.13	2.14

**Fig. 17:**
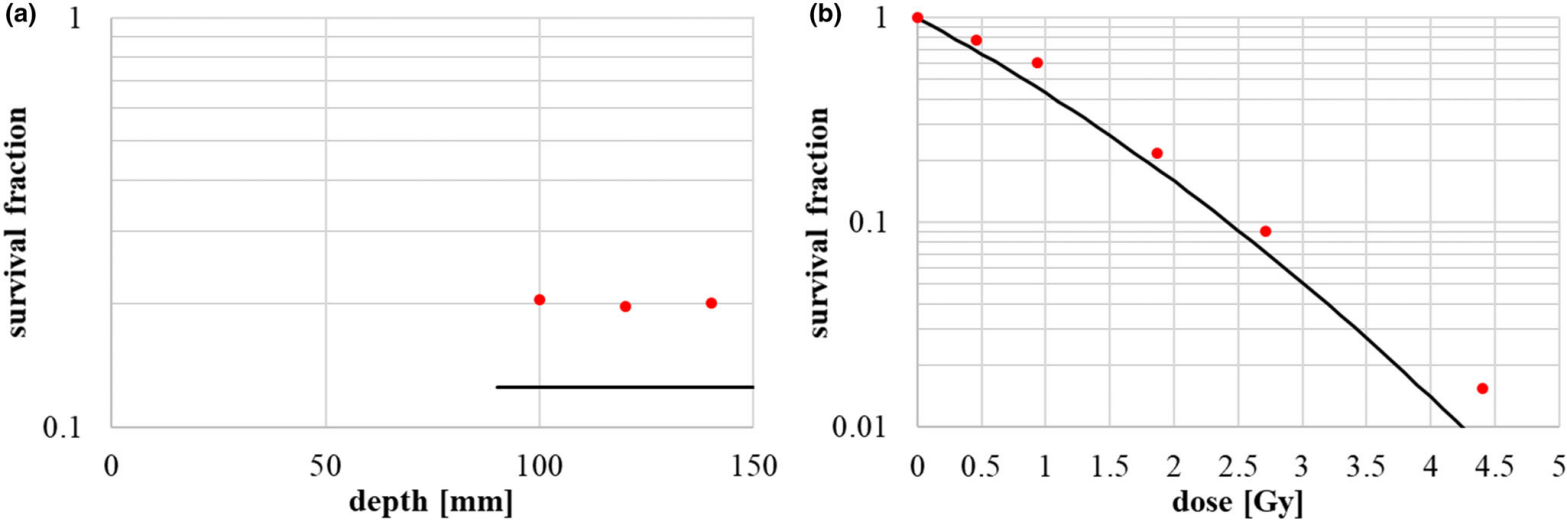
HSG cell survival measurement results: (a) survival in the SOBP flat region, and (b) dose dependency of survival at the center of the SOBP. The red points represent the measurement results and the lines represent the expected survival from calculation.

We next performed the HSG cell irradiation experiment and measured the cell survival in the SOBP region, as shown in Fig. 17. Measurements were taken at three depth points–distal, middle, and proximal–in the 6‐cm SOBP flat region with a range of 15 cm (Fig. 16a), and the observed survival fraction was uniform, as shown in Fig. 17a. The dose dependency of the survival fraction was also measured at the middle of the 6‐cm SOBP, as shown in Fig. 17b. The calculated LQ parameters α and β at the middle were 0.77 [Gy^‐1^] and 0.074 [Gy^‐2^], respectively, and the survival curve is shown by the solid line in Fig. 17b. The calculated survival fraction well‐reproduced the results of the cell survival experiment. The small discrepancy between the calculated results and measurements was probably due to a slight change in the sensitivity of the used HSG cells as described in Discussion in 20.

## 4 DISCUSSION

We have developed physical and biological beam modeling for carbon scanning treatment planning at Osaka HIMAK and reproduced a biological dose distribution compatible with past passive irradiation results. The clinical dose is rather overestimated in the tail region because of the calculation method for the α of fragment particles. Specifically, the calculation of dose‐averaged α*^(He)^* in Eq. (34) via *LETd* may cause overestimation of the biological effect in the tail region. Instead, we could directly score α*^(He)^* in the Geant4 simulation as a function of depth like α*^(C)^*, but we consider this discrepancy tolerable. This is because the fragment α*^(He)^* does not affect the RBE in the SOBP, and overestimation in the tail region is on the side of safety for normal, nontumorous tissue.

In this study, we chose a biological model in which the biological effect of a tumor is always optimized at 10% survival for compatibility with past passive carbon therapy studies, as described in Section 2.B.1. This optimization scheme gives the biological dose calculated by Eq. (31) as 4.03 GyE and the clinical dose by Eq. (32) as 1.46 × 4.03 = 5.88 GyE, so we must introduce a scaling factor between 10% survival and real prescription in treatment planning, because the prescribed dose is specified by the clinical dose to the tumor and the organ at risk (OAR). This scaling factor is introduced as

(41) $$F_{\mathrm{scale}}=\frac{D_{\mathrm{pres}}}{d_{clin,i}\left(S=10\%\right)}≅\frac{D_{\mathrm{pres}}}{5.88\left[GyE\right]},$$

where *D_pres_* denotes the prescribed dose to the target in the treatment planning software, and *d_clin,i_(S = 10%)* is the clinical dose at 10% survival, which is always 5.88 GyE. As for the OAR dose restriction, the goal effect of the OAR is calculated using this scale factor *F_scale_* to preserve the ratio of the prescribed dose to the tumor and the OAR dose constraint:

(42) $$E_{\mathrm{OAR}}=\alpha_{X}\left(\frac{D_{\mathrm{OAR}}}{1.46\;\times \;F_{\mathrm{scale}}}\right)+\beta_{X}{\left(\frac{D_{\mathrm{OAR}}}{1.46\;\times \;F_{\mathrm{scale}}}\right)}^{2},$$

where *D_oar_* is the dose constraint. After dose calculation under the 10% survival condition, this scaling factor is again applied to calculate the clinical dose, physical dose, and the number of particles at each spot, *w_j_*:

(43) $$\begin{array}{c} w_{j}=F_{\mathrm{scale}}\times w_{j}\left(S=10\%\right),\\ d_{i}=F_{\mathrm{scale}}\times d_{i}\left(S=10\%\right),\\ d_{clin,i}=F_{\mathrm{scale}}\times d_{clin,i}\left(S=10\text{\textbackslash{}\%}\;\right), \end{array}$$

where *w_j_(S = 10%)* and *d_i_(S = 10%)* are the number of particles of beam *j* and the physical dose at point *i*, respectively, under the condition of 10% survival. This is a rather complicated process, as when the user scales the MU value. We have to remember this scaling amount for the MU value, because *F_scale_* in Eq. (41) is required for optimization and dose calculation. The disadvantage of this method is that the spot file describing the irradiation points (x and y positions) and the MUs of each spot and the scaling factor must be recorded as a set; otherwise, the spot file cannot reproduce the clinical dose correctly.

We here consider multi‐field irradiation by summing the dose distribution of single‐field optimization. Multifield irradiation is effective to reduce the dose to normal tissue. In carbon passive irradiation, multifield irradiation was performed by adding dose distributions which are each optimized for a survival level of 10%. This is based on the assumption that survival curve in the tumor is dominated by only α‐term of LQ model and neglecting the influence of β‐term. The same scheme of multifield irradiation is possible in scanning irradiation because the biological model is the same.

## 5 CONCLUSION

We have successfully completed physical and biological beam modeling for carbon beam scanning treatment at the Osaka Heavy Ion Therapy Center (Osaka HIMAK) facility. The physical dose calculation was based on a triple Gaussian pencil‐beam algorithm. In this approach, the first component, corresponding to incident ^12^C particles, was modeled by a Monte Carlo simulation and dose measurement using a large‐area ionization chamber and fluorescent screen monitors. In contrast, the second and third components, which describe the fragment halo, were modeled by analytical functions reflecting the results of frame pattern dose measurements. The calculation accuracy of the absolute physical dose was verified to be within ±2% in comparison to volume irradiation dose measurements. Then, for biological dose modeling, we extended a passive ridge‐filter design method to carbon scanning treatment, and we obtained radiation quality LET data and a dose‐averaged LQ parameter α for carbon by Monte Carlo simulation. The RBE in the middle of an SOBP reproduced that of passive dose distribution results to within ±1.5%. Our treatment planning software is currently in clinical use for designing treatment fields with single‐field optimization, and clinical commissioning for multifield optimization irradiation is now underway.

## CONFLICTS OF INTEREST

The authors, Shinichiro Fujitaka, Yusuke Fujii, Hideaki Nihongi, and Satoshi Nakayama are employee of Hitachi, Ltd.

## AUTHOR CONTRIBUTIONS

Shinichiro Fujitaka: Experimental data analysis, investigation, writing original draft preparation. Yusuke Fujii: Experimental data analysis, investigation. Hideaki Nihongi: Experimental data arrangement, analysis. Satoshi Nakayama: Experimental data arrangement, analysis. Masaaki Takashina: Experimental data acquisition, analysis, validation. Noriaki Hamatani: Experimental data acquisition, analysis, validation. Toshiro Tsubouchi: Experimental data acquisition, analysis, validation. Masashi Yagi: Experimental data acquisition, analysis, validation, reviewing original draft. Kazumasa Minami: Biological data acquisition, analysis, writing original draft preparation. Kazuhiko Ogawa: Research direction, supervision. Junetsu Mizoe: Research direction, supervision. Tatsuaki Kanai: Research direction, supervision, methodology proposal, validation, reviewing and editing draft.

## Figures and Tables

**Fig. A1:**
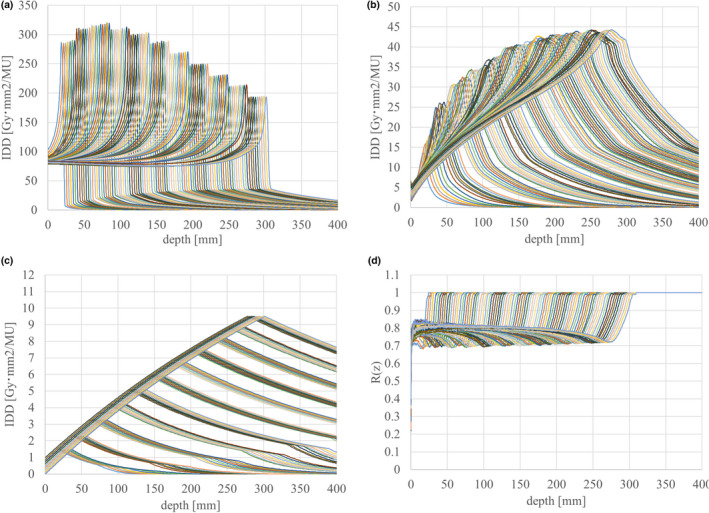
Registered IDD data used in this study: (a) *IDD_total_ (IDD_1_+IDD_2_+IDD_3_)*, (b) *IDD_frag_ (IDD_2_+IDD_3_)*, (c) *IDD_3_*, and (d) ratio of *IDD_frag_* with and without carbon isotopes.

**Fig. A2:**
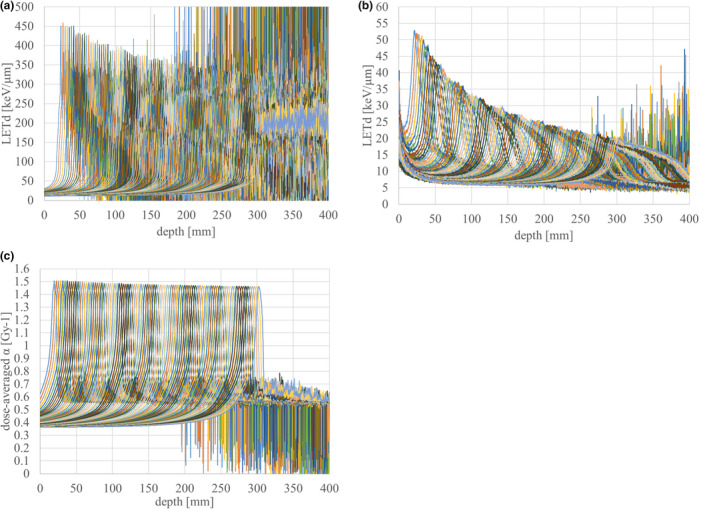
Registered dose‐averaged LET and LQ parameter data used in this study: (a) carbon *LET_C_*, (b) fragment *LET_frag_*, and (c) dose‐averaged carbon *α*.

**Fig. A3:**
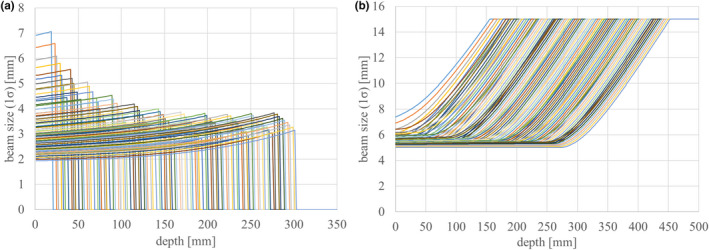
Beam sizes of the (a) first and (b) second components. The beam size of the third component is constant at 25 mm (1σ), regardless of the depth and beam energy.

## Data Availability

Research data are not shared, because the data of this study are obtained from openly available Monte‐Carlo calculation programs and commercially available dosimetric equipments and we think sufficient reproducibility is ensured.

## 1

This appendix gives the registered beam data used in this study. Figures A1, A2, and A3 show the IDD data, LET data and LQ parameters, and beam size data respectively.
